# Supplementary material for: The interoceptive hippocampus: Mouse brain endocrine receptor expression highlights a dentate gyrus (DG)–cornu ammonis (CA) challenge–sufficiency axis
Source: PLoS One. 2020 Jan 15;15(1):e0227575. doi: 10.1371/journal.pone.0227575 (PMC6961916; doi:10.1371/journal.pone.0227575)
Supplement: S1 Appendix — Supplementary tables A–H. (PDF) [file pone.0227575.s001.pdf]

Appendix

SUPPLEMENTARY DATA ONLINE

The interoceptive hippocampus: mouse brain endocrine receptor expression highlights a dentate gyrus (DG)–cornu ammonis (CA) challenge–sufficiency axis

Richard Lathe, Sheena Singadia, Crispin Jordan, and Gernot Riedel

Supplementary data

Table A: List of 250 endocrine receptors studied in this work.

Table B: Primary endocrine receptor gene expression data.

Table C: Primary hippocampal endocrine receptor gene expression data.

Table D: Pairwise correlation analysis.

Table E: List of informative genes.

Table F: Comparison of Allen Brain Atlas and HippoSeq expression data.

Table G: Expression patterns of additional informative receptors confirms the challenge/sufficiency axis.

Table H: Compilation of ligand effects on LTP and neurogenesis.

Table A. List of Endocrine Receptors Analyzed

	**RECEPTOR**
1	ACVR1
2	ACVR1B
3	ACVR1C
4	ACVR2A
5	ACVR2B
6	ACVRL1
7	ADIPOR1
8	ADIPOR2
9	ADORA1
10	AGTR1A
11	AHR
12	AMHR2
13	AR
14	BMPR1B
15	CALCR
16	CALCRL
17	CASR
18	CCKAR
19	CCKBR
20	CCR1
21	CCR10
22	CCR2
23	CCR3
24	CCR4
25	CCR5
26	CCR6
27	CCR7
28	CCR8
29	CCR9
30	CD27
31	CD40
32	CNTFR
33	CRHR1
34	CRHR2
35	CRLF1
36	CSF1R
37	CSF2RB
38	CXCR1
39	CXCR2
40	CXCR3
41	CXCR4
42	CXCR5

43	CXCR6
44	CXCR7
45	CYSTR1
46	CYSTR2
47	DNER
48	EBI1
49	EDAR2
50	EDNRA
51	EDNRB
52	EGFEM1
53	EGFR
54	EPOR
55	ERBB3
56	ERBB4
57	ESR1
58	ESR2
59	ESRRA
60	ESRRB
61	ESRRG
62	F2RL1
63	FAS
64	FFAR1
65	FFAR2
66	FFAR3
67	FGFR1
68	FGFR2
69	FGFR3
70	FGFR4
71	FZD1
72	GALR1
73	GALR2
74	GALR3
75	GCGR
76	GFRA2
77	GHR
78	GHRHR
79	GHSR
80	GIPR
81	GLP1R
82	GLP2R
83	GPER
84	GPR12
85	GRPR1
86	HCRTR1
87	HCRTR2

88	IFNAR1
89	IFNAR2
90	IFNGR1
91	IFNGR2
92	IGF1R
93	IGF2R
94	IL10RA
95	IL10RB
96	IL11RA1
97	IL11RA2
98	IL12RB1
99	IL12RB2
100	IL13RA1
101	IL13RA2
102	IL15RA
103	IL17RA
104	IL17RB
105	IL17RC
106	IL17RD
107	IL18R1
108	IL1R1
109	IL23R
110	IL2RA
111	IL2RB
112	IL6R
113	IL7R
114	INSR
115	INSRR
116	KL
117	KLB
118	LCT
119	LDLR
120	LEPR
121	LIFR
122	LNPEP
123	LPAR6
124	LRP1
125	LRP1B
126	LRP2
127	LTB4R1
128	LTB4R2
129	LTBR
130	MAG
131	MAS1
132	MC1R

133	MC2R
134	MC3R
135	MC4R
136	MC5R
137	MCHR1
138	MET
139	MLNR
140	MPL
141	MRGPRA2B
142	MRGPRB1
143	MRGPRB2
144	MRGPRB3
145	MRGPRB4
146	MRGPRB5
147	MRGPRB8
148	MRGPRD
149	MRGPRE
150	MRGPRF
151	MRGPRG
152	MRGPRH
153	MRGPRX1
154	MST1R
155	MTNR1B
156	NECTIN1
157	NECTIN2
158	NECTIN3
159	NGFR
160	NMBR
161	NMU2R
162	NMUR1
163	NMUR2
164	NOD1
165	NOD2
166	NPR1
167	NPR2
168	NPR3
169	NR3C1
170	NR3C2
171	NR4A3
172	NTSR1
173	NTSR2
174	NURR1
175	OGFRL1
176	OPRL1
177	P2RY1

178	P2RY12
179	P2RY2
180	PAQR3
181	PAQR4
182	PAQR5
183	PAQR6
184	PAQR7
185	PAQR8
186	PAQR9
187	PDGFRA
188	PDGFRB
189	PGR
190	PGRMC1
191	PGRMC2
192	PILRA
193	PILRB
194	PLAUR
195	PPARA
196	PPARD
197	PPARG
198	PPYR1
199	PRLR
200	PTAFR
201	PTGIR
202	PTH1R
203	PTHR2
204	PVR
205	RARA
206	RARB
207	RARG
208	RELL1
209	RELL2
210	RELT
211	RORA
212	RORB
213	RXFP1
214	RXFP2
215	RXFP3
216	RXFP4
217	SCTR
218	SLC9A4
219	SORL1
220	SSTR1
221	SSTR2
222	SSTR4

223	SSTR5
224	TACR1
225	TACR2
226	TGFBR1
227	TGFBR2
228	TGFBR3
229	THRA
230	THRB
231	TNFRSF10B
232	TNFRSF11A
233	TNFRSF11B
234	TNFRSF12A
235	TNFRSF13C
236	TNFRSF14
237	TNFRSF19
238	TNFRSF1A
239	TNFRSF21
240	TNFRSF22
241	TNFRSF23
242	TNFRSF25
243	TNFRSF26
244	TNFRSF8
245	TNFRSF9
246	TRHR
247	TSHR
248	TSHRB
249	TTRHR2
250	VDR
251	VIPR1
252	VIPR2
253	VMN1R234

Table B. Primary Endocrine Receptor Expression Data
(ImageJ scanning arbitrary units)

RECEPTOR	HPC	CB	CX
ACVR1	30.51	0.00	2.31
ACVR1B	12.75	2.87	11.34
ACVR1C	0.00	0.00	0.00
ACVR2A	7.22	0.00	0.00
ACVR2B	0.00	0.00	0.00
ACVRL1	0.00	0.00	7.69
ADIPOR1	6.97	3.17	2.77
ADIPOR2	0.00	5.31	0.00
ADORA1	13.72	0.00	0.00
AGTR1A	16.20	15.91	31.26
AHR	0.00	0.00	0.00
AMHR2	0.00	0.00	0.00
AR	2.69	0.00	0.00
BMPR1B	0.00	0.00	0.00
CALCR	0.00	0.00	0.00
CALCRL	0.00	0.00	0.00
CASR	0.00	0.00	0.00
CCKAR	19.52	7.77	8.60
CCKBR	6.14	0.00	13.16
CCR1	27.87	14.40	29.67
CCR10	0.00	0.00	0.00
CCR2	0.00	0.00	0.00
CCR3	0.00	0.00	0.00
CCR4	0.00	0.00	0.00
CCR5	0.00	0.00	0.00
CCR6	0.00	0.00	0.00
CCR7	0.00	0.00	0.00
CCR8	0.00	0.00	0.00
CCR9	0.00	0.00	0.00
CD27	0.00	0.00	0.00
CD40	13.13	0.00	0.00
CNTFR	16.52	24.71	29.26
CRHR1	0.00	15.21	7.70
CRHR2	0.00	0.00	0.00
CRLF1	36.14	0.00	0.00
CSF1R	16.65	10.09	10.43
CSF2RB	0.00	0.00	0.00
CXCR1	0.00	0.00	0.00
CXCR2	0.00	0.00	0.00
CXCR3	0.00	0.00	0.00
CXCR4	0.00	0.00	0.00
CXCR5	0.00	0.00	0.00
CXCR6	0.00	0.00	0.00

CXCR7	0.00	0.00	0.00
CYSTR1	0.00	0.00	0.00
CYSTR2	0.00	0.00	0.00
DNER	24.06	20.25	7.94
EBI1	0.00	0.00	0.00
EDAR2	0.00	0.00	0.00
EDNRA	0.00	0.00	0.00
EDNRB	0.00	12.70	0.00
EGFEM1	18.43	0.00	0.00
EGFR	0.00	0.00	0.00
EPOR	18.43	0.00	13.65
ERBB3	0.00	0.00	0.00
ERBB4	8.52	0.00	10.19
ESR1	0.00	0.00	0.00
ESR2	0.00	0.00	0.00
ESRRA	10.59	11.30	2.11
ESRRB	0.00	0.00	0.00
ESRRG	0.00	0.00	0.00
F2RL1	13.42	0.00	11.51
FAS	0.00	0.00	0.00
FFAR1	0.00	0.00	0.00
FFAR2	0.00	0.00	0.00
FFAR3	0.00	0.00	0.00
FGFR1	22.33	9.85	0.00
FGFR2	0.00	9.73	6.47
FGFR3	2.29	5.90	2.94
FGFR4	0.00	0.00	0.00
FZD1	11.84	9.96	1.53
GALR1	0.00	0.00	0.00
GALR2	0.00	0.00	0.00
GALR3	2.61	5.50	9.72
GCGR	0.00	0.00	0.00
GFRA2	1.03	7.23	24.50
GHR	0.00	0.00	0.00
GHRHR	0.00	0.00	0.00
GHSR	0.00	0.00	0.00
GIPR	0.00	0.00	0.00
GLP1R	0.00	0.00	0.00
GLP2R	0.00	0.00	11.56
GPER	0.00	0.00	0.00
GPR12	10.88	0.00	0.00
GRPR1	0.00	0.00	0.00
HCRTR1	6.42	0.00	0.00
HCRTR2	0.00	0.00	0.00
IFNAR1	26.14	26.54	35.09
IFNAR2	0.00	0.00	0.00
IFNGR1	0.00	0.00	4.28
IFNGR2	13.97	0.00	0.00

IGF1R	21.03	9.04	1.57
IGF2R	7.33	5.45	10.16
IL10RA	0.00	0.00	0.00
IL10RB	47.48	13.47	29.79
IL11RA1	6.33	1.61	3.20
IL11RA2	0.00	0.00	8.66
IL12RB1	0.00	0.00	0.00
IL12RB2	0.00	0.00	0.00
IL13RA1	0.00	0.00	0.00
IL13RA2	0.00	0.00	0.00
IL15RA	0.00	0.00	0.00
IL17RA	0.00	0.00	11.34
IL17RB	0.00	0.00	0.00
IL17RC	0.00	0.00	0.00
IL17RD	32.79	0.10	9.18
IL18R1	0.00	0.00	0.00
IL1R1	11.60	0.00	0.00
IL23R	0.00	0.00	0.00
IL2RA	0.00	0.00	0.00
IL2RB	12.17	2.47	0.10
IL6R	0.00	0.00	0.00
IL7R	0.00	0.00	0.00
INSR	0.00	0.00	0.00
INSRR	0.00	0.00	0.00
KL	28.77	7.80	5.11
KLB	0.00	0.00	0.00
LCT	23.65	0.00	0.00
LDLR	0.00	0.00	0.00
LEPR	0.00	0.00	0.00
LIFR	10.80	3.31	11.91
LNPEP	39.89	48.81	26.99
LPAR6	0.00	0.00	0.00
LRP1	34.69	8.75	23.84
LRP1B	8.87	0.00	1.01
LRP2	0.00	0.00	0.00
LTB4R1	0.00	0.00	0.00
LTB4R2	0.00	0.00	0.00
LTBR	17.55	8.67	7.51
MAG	8.36	17.47	17.59
MAS1	24.37	0.00	0.00
MC1R	0.00	0.00	0.00
MC2R	12.03	0.00	0.00
MC3R	0.00	0.00	0.00
MC4R	0.00	0.00	0.00
MC5R	0.00	0.00	0.00
MCHR1	0.00	0.00	0.00
MET	0.00	0.00	0.00

MLNR	0.00	0.00	0.00
MPL	0.00	0.00	0.00
MRGPRA2B	0.00	0.00	0.00
MRGPRB1	0.00	0.00	0.00
MRGPRB2	0.00	0.00	0.00
MRGPRB3	0.00	0.00	0.00
MRGPRB4	0.00	0.00	0.00
MRGPRB5	0.00	0.00	0.00
MRGPRB8	0.00	0.00	0.00
MRGPRD	0.00	0.00	0.00
MRGPRE	0.00	0.00	0.00
MRGPRF	0.00	0.00	0.00
MRGPRG	0.00	0.00	0.00
MRGPRH	0.00	0.00	0.00
MRGPRX1	0.00	0.00	0.00
MST1R	0.00	0.00	0.00
MTNR1B	0.00	0.00	0.00
NECTIN1	38.35	13.10	7.10
NECTIN2	17.35	8.03	9.04
NECTIN3	21.95	0.00	23.77
NGFR	0.00	0.00	0.00
NMBR	0.00	0.00	0.00
NMU2R	0.00	0.00	0.00
NMUR1	0.00	0.00	0.00
NMUR2	0.00	0.00	0.00
NOD1	0.00	0.00	0.00
NOD2	0.00	0.00	0.00
NPR1	0.00	9.20	0.00
NPR2	25.20	12.88	8.88
NPR3	5.08	0.00	2.20
NR3C1	12.40	0.00	3.83
NR3C2	11.13	0.00	0.00
NR4A3	17.72	0.00	0.00
NTSR1	0.00	0.00	0.00
NTSR2	0.00	0.00	0.00
NURR1	11.31	23.54	13.67
OGFRL1	13.01	7.81	1.33
OPRL1	10.77	0.00	6.52
P2RY1	0.00	0.00	0.00
P2RY12	2.05	0.00	3.12
P2RY2	4.21	0.00	0.00
PAQR3	0.00	0.00	0.00
PAQR4	12.47	6.97	6.75
PAQR5	0.00	0.00	0.00
PAQR6	0.00	6.38	0.00
PAQR7	22.27	11.65	28.14
PAQR8	14.38	10.25	1.60
PAQR9	24.86	0.00	1.13

PDGFRA	3.50	2.82	4.99
PDGFRB	1.50	2.24	2.27
PGR	0.00	0.00	0.00
PGRMC1	19.39	0.90	2.05
PGRMC2	25.26	9.84	12.94
PILRA	0.00	0.00	0.00
PILRB	0.00	0.00	0.00
PLAUR	0.00	0.00	0.00
PPARA	0.00	0.00	0.00
PPARD	0.00	0.00	0.00
PPARG	47.40	18.18	32.09
PPYR1	0.00	0.00	0.00
PRLR	0.00	0.00	0.00
PTAFR	0.00	0.00	0.00
PTGIR	11.36	25.40	2.83
PTH1R	0.00	0.00	0.00
PTHR2	0.00	0.00	0.00
PVR	36.91	46.29	36.21
RARA	12.70	6.86	1.20
RARB	0.00	0.00	0.00
RARG	24.58	0.00	11.18
RELL1	0.00	0.00	0.00
RELL2	0.00	0.00	0.00
RELT	0.00	0.00	0.00
RORA	2.51	21.62	19.72
RORB	0.00	0.00	24.62
RXFP1	0.92	0.00	4.61
RXFP2	0.00	0.00	0.00
RXFP3	3.86	0.00	0.00
RXFP4	0.00	0.00	0.00
SCTR	0.00	0.00	0.00
SLC9A4	11.99	0.00	0.56
SORL1	50.64	18.79	36.91
SSTR1	0.00	0.00	0.00
SSTR2	0.00	0.00	5.47
SSTR4	19.61	0.00	4.64
SSTR5	0.00	0.00	0.00
TACR1	0.00	0.00	0.00
TACR2	0.00	0.00	0.00
TGFBR1	0.00	0.00	0.00
TGFBR2	0.00	1.42	5.42
TGFBR3	21.02	7.65	3.75
THRA	31.17	0.00	16.20
THRB	17.55	0.00	11.70
TNFRSF10B	0.00	0.00	0.00
TNFRSF11A	0.00	0.00	0.00
TNFRSF11B	0.00	0.00	0.00
TNFRSF12A	0.00	0.00	0.00

TNFRSF13C	0.00	0.00	0.00
TNFRSF14	0.00	0.00	0.00
TNFRSF19	11.17	0.00	4.36
TNFRSF1A	0.00	0.00	0.00
TNFRSF21	0.00	0.00	0.00
TNFRSF22	0.00	0.00	0.00
TNFRSF23	0.00	0.00	0.00
TNFRSF25	19.71	0.00	3.87
TNFRSF26	0.00	0.00	0.00
TNFRSF8	0.00	0.00	0.00
TNFRSF9	0.00	0.00	0.00
TRHR	0.00	0.00	0.00
TSHR	0.00	0.00	0.00
TSHRB	0.00	0.00	0.00
TTRHR2	0.00	0.00	0.00
VDR	0.00	0.00	0.00
VIPR1	11.21	0.00	8.86
VIPR2	0.00	0.00	0.00
VMN1R234	23.15	0.00	0.00

Table C. Primary hippocampal subregion informative receptor expression data

Receptor	CA1	CA2	CA3	DG	CA1	CA2	CA3	DG	
IL1R1	0	0	0	1	0	0	0	100	A
MAS1	0.037	0.44	0.464	1	3.7	44	46.4	100	
CRLF1	0.265	0.052	0.392	1	26.5	5.2	39.2	100	
PPARG	0.282	0.367	0.417	1	28.2	36.7	41.7	100	
IL17RD	0.3	0.2	0.2	1	30	20	20	100	
TNFRSF25	0.361	0.322	0.286	1	36.1	32.2	28.6	100	
CCR1	0.526	0.505	0.57	1	52.6	50.5	57	100	
TGFBR3	0.565	0.328	0.442	1	56.5	32.8	44.2	100	
IFNAR1	0.6	0.6	0.6	1	60	60	60	100	
IL10RB	0.788	0.71	0.518	1	78.8	71	51.8	100	
NR3C2	0.685	0.763	0.671	1	68.5	76.3	67.1	100	
VIPR1	0.626	0.183	0.896	1	62.6	18.3	89.6	100	
F2RL1	0.589	1	0.067	0.879	58.9	100	6.7	87.9	
TNFRSF21	0.767	0.849	0.893	1	76.7	84.9	89.3	100	
AGTR1A	0.884	0.836	0.77	1	88.4	83.6	77	100	
TNFRSF19	1	0.509	0.97	0.846	100	50.9	97	84.6	
IL2RB	0.932	0.168	1	0.77	93.2	16.8	100	77	
PGRMC2	0.377	0.323	1	0.276	37.7	32.3	100	27.6	B
THRA	0.432	0.658	1	0.244	43.2	65.8	100	24.4	
RORA	0.574	1	0.915	0.246	57.4	100	91.5	24.6	
CCKAR	0.8	1	0.91	0.75	80	100	91	75	
FGFR1	0.78	1	0.884	0.53	78	100	88.4	53	
FGFR3	0.1	1	0.467	0.033	10	100	46.7	3.3	
RARG	1	0.697	0.601	0.599	100	69.7	60.1	59.9	
ESRRA	1	0.309	0.58	0.638	100	30.9	58	63.8	
PGRMC1	1	0.485	0.749	0.592	100	48.5	74.9	59.2	

RARA	1	0.552	0.54	0.311	100	55.2	54	31.1
THRB	1	0.526	0.237	0.203	100	52.6	23.7	20.3
KL	1	0.039	0.221	0.2	100	3.9	22.1	20
SSTR4	1	0.293	0	0	100	29.3	0	0
CCKBR	1	0	0	0	100	0	0	0
AR	1	0	0	0	100	0	0	0

Table D. Cross-Correlation Analysis

95% CI values for correlation of normalized (and arcsine square root transformed) gene expression between regions CA1, CA2, CA3, and DG, showing significant positive correlations between CA2 and CA3, and significant negative correlations between CA1 and DG (Bold Font).[a]

	CA2	CA3	DG
CA1	−0.1652, 0.3194	−0.2902, 0.2440	**−0.5067, −0.0990**
CA2		**0.1991, 0.6087**	−0.1730, 0.2813
CA3			−0.3727, 0.1771

[a]In most comparisons, non-normalized gene expression did not differ significantly among regions CA1, CA2, CA3 and DG (paired t tests (df = 85): CA1 and CA2: $t = 2.5221$, $P = 0.01353$; CA1 and CA3: $t = 1.2136$, $P = 0.2283$; CA1 and DG: $t = -1.6319$, $P = 0.1064$; CA2 and CA3: $t = -1.2556$, $P = 0.2127$; CA2 and DG: $t = -3.3113$, $P = 0.001364$; CA3 and DG: $t = -2.4651$, $P = 0.01571$); only the difference between CA2 and DG remained significant after accounting for multiple comparisons, with higher gene expression in DG.

Table E. List of 'Informative' Endocrine Receptors[a]

Receptor	Full name	Known ligands
IL1R1	Interleukin 1 receptor type 1	IL-1α, IL-1β, IL-1RN
MAS1	MAS1 proto-oncogene GPCR	Angiotensin (1–7)
CRLF1	Cytokine receptor-like factor 1	Cardiotrophin-like cytokine (CLC); the CRLF1/CLC complex activates the ciliary neurotrophic factor receptor (CNTFR)
PPARG	Peroxisome proliferator-activated receptor γ	Prostaglandin J2 (15d-PGJ2); 5-oxo-15(S)-HETE and 5-oxo-ETE
IL17RD	Interleukin 17 receptor D	IL-17
TNFRSF25	TNF receptor superfamily 25	TNF-α/TNFSF12
CCR1	C-C motif chemokine receptor 1	Macrophage inflammatory protein 1α (MIP-1α), RANTES, MCP-3, and MPIF-1.
TGFBR3	Transforming growth factor β receptor 3	TGF-β superfamily members
IFNAR1	Inferferon α/β receptor subunit 1	Type I interferons, including IFNA, IFNB1, and IFNW1
IL10RB	Interleukin 10 receptor subunit β	IL-10, IL-22, IL-26, IL-28, and IFN-L1
NR3C2	Nuclear receptor subfamily 3C2; mineralocorticoid receptor (MR)	In the absence of HSD11B expression, corticosterone (mouse)/cortisol (human) is the principal ligand (Box 1)
VIPR1	Vasoactive intestinal polypeptide receptor 1	VIP
F2RL1	F2R-like trypsin receptor 1/protease-activated receptor PAR2	Trypsin/thrombin; activated by local proteolysis
TNFRSF21	TNF receptor superfamily 21	TNF superfamily members
AGTR1A	Angiotensin II receptor type 1	Angiotensin II (Ang 1–8)
TNFRSF19	TNF receptor superfamily 19	TNF superfamily members
IL2RB	Interleukin 2 receptor subunit β	IL-2
PGRMC2	Progesterone receptor membrane component 2	Progesterone/possible sigma-2 receptor by analogy to PGRMC1
THRA	Thyroid hormone receptor α	Thyroid hormone (triiodothyronine, T3, and thyroxine, T4)
RORA	RAR-related orphan receptor A	Endogenous ligand presumed to be related to retinoic acid; potential ligands include cholesterols and vitamin D derivatives
CCKAR	Cholecystokinin A receptor	Non-sulfated members of the cholecystokinin (CCK) family
FGFR1	Fibroblast growth factor receptor 1	Inferred to respond to endocrine FGFs including FGF19, FGF21, and FGF23 (Box 1)
FGFR3	Fibroblast growth factor receptor 3	Inferred to respond to endocrine FGFs such as FGF19, FGF21, and FGF23 (Box 1)
RARG	Retinoic acid receptor γ	All-*trans* retinoic acid, 9-*cis* retinoic acid
ESRRA	Estrogen-related receptor α	Estrogen-related molecules (Box 1), cholesterols, possibly farnesyl pyrophosphate
PGRMC1	Progesterone receptor membrane component 1	Progesterone; sigma-2 receptor (e.g., haloperidol)
RARA	Retinoic acid receptor α	All-*trans* retinoic acid, 9-*cis* retinoic acid
THRB	Thyroid hormone receptor β	Thyroid hormone (triiodothyronine, T3, and thyroxine, T4)
KL	Klotho	FGF coreceptor; inferred to respond to endocrine FGFs including FGF19, FGF21, and FGF23 (Box 1)
SSTR4	Somatostatin receptor 4	Somatostatin peptides (SST-14, SST-28)
CCKBR	Cholecystokinin B receptor	Gastrin and cholecystokinin (CCK)
AR	Androgen receptor; nuclear receptor superfamily 3C4	Testosterone, dihydrotestosterone, modified estrogens (e.g., androstenediol; Box 1)

[a]Receptor order is a gradient, with DG-expressed receptors at the top of the table, and CA-expressed receptors at the foot.

Table F. HippoSeq Analysis of Differential Hippocampal Gene Expression; Comparison of Allen Brain Atlas (ABA) and HippoSeq Analyses of Gene Expression in Mouse Hippocampus

A. DG versus CA Expression Ratios for Informative Genes (Hipposeq)[a,b]

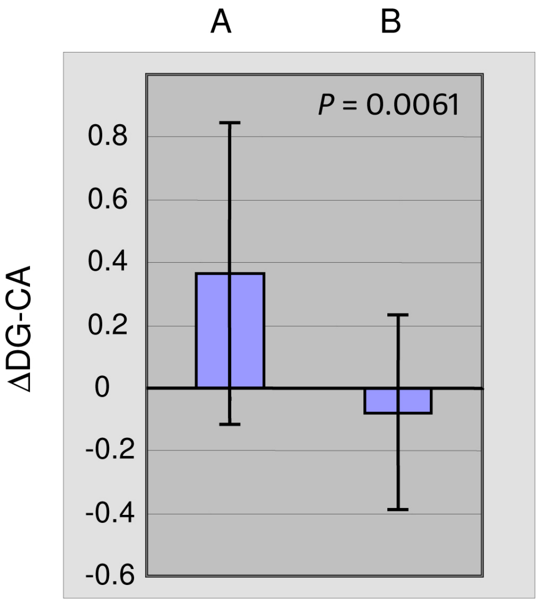

A = 'challenge; B = 'sufficiency'

[a]For each gene, expression data for DG and CA regions were downloaded as fragments per kilobase million (FPKM) from the HippSeq website. Mean CA and DG FPKM values were calculated, 1.0 was added to each value to avoid div0 errors, values were converted to log10, and the ratio of DG versus CA expression calculated as the difference between the log(DG) and log(CA) values (i.e., ΔDG−CA, where values above 1.0 indicate enrichment in DG, and values below 1.0 indicate enrichment in CA regions) as before. For statistical analysis (Welch's *t* test), means and SDs of the ΔDG−CA values were calculated. Because we do not rely on these data for our central analysis, the random permutation refinement (Methods) was not performed.

[b]Although the overall patterns were conserved, we draw attention to discordances between ABA and Hipposeq for a small number of genes For example, Hipposeq detected significant expression of *Fgrr3* and *Rora* in DG; conversely, *Tnfrsf21* expression was seen in CA regions. These differences could potentially reflect differences in the methodologies employed (Table S6B).

B. Methodologies: ABA versus HippoSeq

	**ABA**	**HippoSeq**
Method	*In situ* hybridization with coding-region probes following formalin fixation	Deep sequencing of transgenically selected marked cells followed by computer matching
Mouse strain	Mixture<br>129S1/SvlmJ<br>C57BL/6J<br>CAST/EiJ<br>DBA/2J<br>PWD/PhJ<br>SPRET/EiJ<br>WSB/EiJ	C57BL/6
Sex	Mixture M/F	Mixture M/F
Age	4 Weeks	Postnatal days P25–P32
Hippocampus	Gradient; dorsal selected	Dorsal selected
Phase of light/dark cycle	8 am–noon	Mid light cycle
Number of repeats for one receptor	In most cases, single animals for each probe	100 cells per animal, three animals per receptor
n (animals)	*n* = 253	*n* = 3 for each region (pooled); for 7 regions *n* = 21
Output	*In situ* hybridization intensity, single probe (or small number), amplitude score (arbitrary units via ImageJ)	FPKM sequence reads; single transgenic expression tag (note: high inter-sample variation in FPKM reads)

Table G. Expression Patterns of Additional Informative Receptors Confirms the Challenge/Sufficiency Axis

Full name	Gene	Challenge/ sufficiency	Hippocampal expression[a]
Interleukin 6 receptor (IL-6 is induced by infection)	*Il6r*	Challenge	DG >> CA regions
Growth hormone secretagogue receptor (ghrelin receptor); ghrelin stimulates hunger/food intake (indicator of deficit)	*Ghsr*	Challenge	DG >> CA regions
Opioid growth factor receptor (Met5 encephalin receptor; inhibits cell growth)	*Ogfr*	Challenge	DG >> CA regions
Irisin receptor (irisin stimulates gluconeogenesis – indicator of deficit). (Note, may have different functions in mouse and human)	*Itgav*	Challenge	DG >> CA regions
Somatostatin/corticostatin receptor; function in stress responses (Box S1)	*Sstr2*	Challenge	DG >> CA regions
Leptin receptor (leptin reduces food intake – indicator of sufficiency)	*Lepr*	Sufficiency	CA1 ~ CA3 ~ DG
Glucagon-like peptide 1 receptor (GLP1 stimulates insulin secretion and glucose uptake)	*Glp1r*	Sufficiency	CA3/CA1 >> DG

[a]Data: Allen Brain Atlas (ABA) and/or HippoSeq. For *Il6r*, *Ghsr*, and *Sstr2* expression was below the limit of detection in ABA, but HippoSeq confirmed restricted expression principally in DG.

Table H. Ligand Effects on LTP and Neurogenesis

Table S8. Literature survey of ligand/receptor effects on long-term potentiation (LTP) and neurogenesis

Receptor	Full name	Ligand(s)	Effects of activation on LTP in DG	Effects of activation on LTP in CA1	Refs	Effects on neurogenesis in DG	Refs
IL1R1	Interleukin 1 receptor type 1	IL-1α	↓		(1)	↓	(6)
		IL1-β	↓	↓	(2-5)		
MAS1	MAS1 proto-oncogene GPCR	Angiotensin 1–7		↑[d]	(7)	↓	(8)
CRLF1	Cytokine receptor-like factor 1	Cardiotrophin-like cytokine (CLC); the CRLF1/CLC complex activates the ciliary neurotrophic factor receptor (CNTFR)			N/L	↑	(9-11)
PPARG	Peroxisome proliferator-activated receptor gamma	Prostaglandin J2 (15d-PGJ2); 5-oxo-15(S)-HETE and 5-oxo-ETE		NE	(12)	↑ (ld)<br>↓ (hd)	(13)
IL17RD	Interleukin 17 receptor D	IL-17			N/L	↓	(14)
TNFRSF25	TNF receptor superfamily 25	TNFSF12 (TNFα)	↓	NE	(15,16)<br>(17)	↓	(18)

CCR1	C-C motif chemokine receptor 1	Macrophage inflammatory protein 1α (MIP-1α – also known as CCL3)		↓	(19)	N/L	
		RANTES		NE	(20)		
		MCP-3 and MPIF-1			N/L		
TGFBR3	Transforming growth factor beta receptor 3	TGF-β superfamily members	↓	↑	(21) (22)	↑	(23;24)
IFNAR1	Inferferon α/β receptor subunit 1	Type I interferons, including IFNA, IFNB1, and IFNW1		↓	(25)	↓	(26)
IL10RB	Interleukin 10 receptor subunit β	IL10, IL22, IL26, IL28, and IFNL1	↓		(27)		N/L
NR3C2	Nuclear receptor subfamily 3C2; mineralocorticoid receptor	In the absence of HSD11B expression, corticosterone (mouse)/cortisol (human) is the principal ligand (see Box 1)	↓	↓	(28) (29;30)	↓	(31;32)
VIPR1	Vasoactive intestinal polypeptide receptor 1	VIP		↓	(33)	↓	(34;35)
F2RL1	F2R-like trypsin receptor 1/protease-activated receptor PAR2	Trypsin/thrombin; activated by local proteolysis		↑	(36;37;38)	↓	(39)
TNFRSF21	TNF receptor superfamily 21	TNF superfamily members			See TNFRSF25		N/L
AGTR1A	Angiotensin II receptor type 1	Angiotensin II (Ang 1–8)	↓		(40-45)	↑	(46;47)

TNFRSF19	TNF receptor superfamily 19	TNF superfamily members			See TNFRSF25		N/L
IL2RB	Interleukin 2 receptor subunit beta	IL-2		↓	(48)	↓	(49)
PGRMC2	Progesterone receptor membrane component 2	Progesterone/possible sigma-2 receptor by analogy to PGRMC1		↓	(50)	↑	(51;52)
THRA	Thyroid hormone receptor alpha	Thyroid hormone (triiodothyronine, T3, and thyroxine, T4)	↓	↑	(53-55) (56)	↑	(57)
RORA	RAR related orphan receptor A	Endogenous ligand presumed to be related to retinoic acid; potential ligands include cholesterols and vitamin D derivatives			N/L		N/L
CCKAR	Cholecystokinin A receptor	Non-sulfated members of the cholecystokinin (CCK) family		↑ NE	(58) (59)	↑	(60;61)
FGFR1	Fibroblast growth factor receptor 1	Inferred to respond to endocrine FGFs including FGF19, FGF21, and FGF23 (Box 1)	↑		(62)	↑	(62;63)
FGFR3	Fibroblast growth factor receptor 3	Inferred to respond to endocrine FGFs such as FGF19, FGF21, and FGF23 (Box 1)			N/L (for FGF, see above)	↑	(63)
RARG	Retinoic acid receptor gamma	All-*trans* retinoic acid, 9-*cis* retinoic acid		NE	(64)		N/L

ESRRA	Estrogen-related receptor alpha	Estrogen-related molecules (Box 1), cholesterols, possibly farnesyl pyrophosphate		↑	(65;66)		N/L
PGRMC1	Progesterone receptor membrane component 1	Progesterone; sigma-2 receptor (e.g., haloperidol)			See above for PGRMC2	↑	(67)
RARA	Retinoic acid receptor alpha	All-*trans* retinoic acid, 9-*cis* retinoic acid		↑	(68)	↑	(69)
THRB	Thyroid hormone receptor beta	Thyroid hormone (triiodothyronine, T3, and thyroxine, T4)			N/L	↑	(70)
KL	Klotho	FGF coreceptor; inferred to respond to endocrine FGFs including FGF19, FGF21, and FGF23 (Box 1)		↑	(71)	↑	(72)
SSTR4	Somatostatin receptor 4	Somatostatin peptides (SST-14, SST-28)	↓[e]	↑[e]	(73)<br>(74)		N/L
CCKBR	Cholecystokinin B receptor	Gastrin and cholecystokinin (CCK)		↑	(58;59)		N/L
AR	Androgen receptor; nuclear receptor superfamily 3C4	Testosterone, dihydrotestosterone (androstenediol; Box 1)	↑	↑	(75)<br>(76;77)	↑	(78-80)

[a]Table summarizes the list of putative agonists for the informative receptors and the effects of ligands that activate the receptors. Data on long-term potentiation as a proxy for synaptic plasticity (other forms were neglected here; also, no discrimination between early and late phase LTP was performed) are mainly derived from work on rat both in vivo and in brain slices; they are extrapolated to murine function as data on mouse LTP are relatively scarce. Only positive data are mentioned, if no arrow is shown, no literature is available.

[b]Receptor order is a gradient as in Figure 3, with DG-expressed receptors at the top of the table, and CA-expressed receptors at the foot.

[c]Key: ↑ indicates enhancement, ↓ indicates reduction/suppression of activity; N/L, no available literature; NE, no effect reported by cited work; ld, low dose; hd, high dose.

[d]See also AGTR1A.

[e]Note difficulty to differentiate between neuronally released somatostatin from interneurons and exogenous somatostatin from blood.

Literature cited

(1) Griffin R, Nally R, Nolan Y, McCartney Y, Linden J, Lynch MA (2006) The age-related attenuation in long-term potentiation is associated with microglial activation. J Neurochem. 99(4):1263-72.

(2) Coogan, AN, O'Neill, LA, O'Connor, JJ. (1999) The P38 mitogen-activated protein kinase inhibitor SB203580 antagonizes the inhibitory effects of interleukin-1beta on long-term potentiation in the rat dentate gyrus in vitro. Neuroscience 93, 57–69

(3) Coogan A, O'Connor JJ (1997) Inhibition of NMDA receptor-mediated synaptic transmission in the rat dentate gyrus *in vitro* by IL-1b. *NeuroReport* 8, 2107–2110.

(4) Bellinger FP, Madamba S, Siggins GR. (1993) Interleukin 1 beta inhibits synaptic strength and long-term potentiation in the rat CA1 hippocampus. Brain Res. 19;628(1-2):227-34

(5) Aleph Prieto G, Snigdha S, Baglietto-Vargas D, Smith ED, Berchtold NC, Tong L, Ajami D, LaFerla FM, Rebek J Jr, Cotman CW. (2015) Synapse-specific IL-1 receptor subunit reconfiguration augments vulnerability to IL-1β in the aged hippocampus Proc Natl Acad Sci U S A. 112(36):E5078-87.

(6) Kuzumaki N, Ikegami D, Imai S, Narita M, Tamura R, Yajima M, Suzuki A, Miyashita K, Niikura K, Takeshima H, Ando T, Ushijima T, Suzuki T, Narita M. (2010) Enhanced IL-1beta production in response to the activation of hippocampal glial cells impairs neurogenesis in aged mice. Synapse 64(9):721-8.

(7) Hellner K, Walther T, Schubert M, Albrecht D. (2005) Angiotensin-(1-7) enhances LTP in the hippocampus through the G-protein-coupled receptor Mas. Mol Cell Neurosci. 29(3):427-35.

(8) Freund M, Walther T, von Bohlen U, Halbach O. (2014) Effects of the angiotensin-(1-7) receptor Mas on cell proliferation and on the population of doublecortin positive cells within the dentate gyrus and the piriform cortex. Eur Neuropsychopharmacol. 24(2):302-8.

(9) Kazim SF, Iqbal K. (2016) Neurotrophic factor small-molecule mimetics mediated neuroregeneration and synaptic repair: emerging therapeutic modality for Alzheimer's disease. Mol Neurodegener. 11(1):50.

(10) Emsley JG, Hagg T. (2003) Endogenous and exogenous ciliary neurotrophic factor enhances forebrain neurogenesis in adult mice.Exp Neurol. 183(2):298-310.

(11) Müller S, Chakrapani BP, Schwegler H, Hofmann HD, Kirsch M. (2009) Neurogenesis in the dentate gyrus depends on ciliary neurotrophic factor and signal transducer and activator of transcription 3 signaling. Stem Cells. 27(2):431-41.

(12) Costello DA, O'Leary DM, Herron CE. (2005) Agonists of peroxisome proliferator-activated receptor-gamma attenuate the Abeta-mediated impairment of LTP in the hippocampus in vitro. Neuropharmacology. 49(3):359-66.

(13) Katura T, Moriya T, Nakahata N. (2010) 15-Deoxy-delta 12,14-prostaglandin J2 biphasically regulates the proliferation of mouse

hippocampal neural progenitor cells by modulating the redox state. Mol Pharmacol. 77(4):601-11.

(14) Liu Q, Xin W, He P, Turner D, Yin J, Gan Y, Shi FD, Wu J. (2014) Interleukin-17 inhibits adult hippocampal neurogenesis. Sci Rep. 4:7554.

(15) Curran BP, Murray HJ, O'Connor JJ. (2003) A role for c-Jun N-terminal kinase in the inhibition of long-term potentiation by interleukin-1beta and long-term depression in the rat dentate gyrus in vitro. Neuroscience. 118(2):347-57.

(16) Pickering M, Cumiskey D, O'Connor JJ. (2005) Actions of TNF-alpha on glutamatergic synaptic transmission in the central nervous system. Exp Physiol. 90(5):663-70.

(17) Albensi BC, Mattson MP. (2000) Evidence for the involvement of TNF and NF-kappaB in hippocampal synaptic plasticity. Synapse. 35(2):151-9.

(18) Zhang K, Li YJ, Feng D, Zhang P, Wang YT, Li X, Liu SB, Wu YM, Zhao MG. (2017) Imbalance between TNFα and progranulin contributes to memory impairment and anxiety in sleep-deprived mice. Sci Rep. 7:43594.

(19) Marciniak E, Faivre E, Dutar P, Alves Pires C, Demeyer D, Caillierez R, Laloux C, Buée L, Blum D, Humez S. (2015) The Chemokine MIP-1α/CCL3 impairs mouse hippocampal synaptic transmission, plasticity and memory. Sci Rep. 5:15862.

(20) Mori F, Nisticò R, Nicoletti CG, Zagaglia S, Mandolesi G, Piccinin S, Martino G, Finardi A, Rossini PM, Marfia GA, Furlan R, Centonze D. (2016) RANTES correlates with inflammatory activity and synaptic excitability in multiple sclerosis. Mult Scler. 22(11):1405-1412.

(21) Ikegaya Y, Saito H, Torii K, Nishiyama N. (1997) Activin selectively abolishes hippocampal long-term potentiation induced by weak tetanic stimulation in vivo. Jpn J Pharmacol. 75(1):87-9.

(22) Caraci F, Gulisano W, Guida CA, Impellizzeri AA, Drago F, Puzzo D, Palmeri A.A key role for TGF-β1 in hippocampal synaptic plasticity and memory. Sci Rep. 2015 Jun 10;5:11252.

(23) De Lucia C, Rinchon A, Olmos-Alonso A, Riecken K, Fehse B, Boche D, Perry VH, Gomez-Nicola D. (2016) Microglia regulate hippocampal neurogenesis during chronic neurodegeneration. Brain Behav Immun. 55:179-190.

(24) He Y, Zhang H, Yung A, Villeda SA, Jaeger PA, Olayiwola O, Fainberg N, Wyss-Coray T. (2014) ALK5-dependent TGF-β signaling is a major determinant of late-stage adult neurogenesis.Nat Neurosci. 17(7):943-52.

(25) Mendoza-Fernández V, Andrew RD, Barajas-López C. (2000) Interferon-alpha inhibits long-term potentiation and unmasks a long-term depression in the rat hippocampus. Brain Res. 885(1):14-24.

(26) Kaneko N, Kudo K, Mabuchi T, Takemoto K, Fujimaki K, Wati H, Iguchi H, Tezuka H, Kanba S. (2006) Suppression of cell proliferation by interferon-alpha through interleukin-1 production in adult rat dentate gyrus. Neuropsychopharmacology. 2006 Dec;31(12):2619-26.

(27) Kelly A, Lynch A, Vereker E, Nolan Y, Queenan P, Whittaker E, O'Neill LA, Lynch MA. (2001) The anti-inflammatory cytokine, interleukin (IL)-10, blocks the inhibitory effect of IL-1 beta on long term potentiation. A role for JNK. J Biol Chem. 276(49):45564-72.

(28) Pavlides C, Watanabe Y, McEwen BS. (1993) Effects of glucocorticoids on hippocampal long-term potentiation. Hippocampus. 3(2):183-92.

(29) Rey M, Carlier E, Talmi M, Soumireu-Mourat B. (1994) Corticosterone effects on long-term potentiation in mouse hippocampal slices. Neuroendocrinology. 60(1):36-41.

(30) Zhou J, Zhang F, Zhang Y. (2000) Corticosterone inhibits generation of long-term potentiation in rat hippocampal slice: involvement of brain-derived neurotrophic factor. Brain Res. 885(2):182-91.

(31) Cameron HA, Gould E. (1994) Adult neurogenesis is regulated by adrenal steroids in the dentate gyrus. Neuroscience. 61(2):203-9.

(32) Hellsten J, Wennström M, Mohapel P, Ekdahl CT, Bengzon J, Tingström A. (2002) Electroconvulsive seizures increase hippocampal neurogenesis after chronic corticosterone treatment. Eur J Neurosci. 16(2):283-90.

(33) Cunha-Reis D, Aidil-Carvalho Mde F, Ribeiro JA. (2014) Endogenous inhibition of hippocampal LTD and depotentiation by vasoactive intestinal peptide VPAC1 receptors. Hippocampus. 24(11):1353-63.

(34) Zaben M, Sheward WJ, Shtaya A, Abbosh C, Harmar AJ, Pringle AK, Gray WP. (2009) The neurotransmitter VIP expands the pool of symmetrically dividing postnatal dentate gyrus precursors via VPAC2 receptors or directs them toward a neuronal fate via VPAC1 receptors. Stem Cells. 27(10):2539-51.

(35) Zaben MJ, Gray WP. (2013) Neuropeptides and hippocampal neurogenesis. Neuropeptides. 47(6):431-8.

(36) Maggio N, Itsekson Z, Dominissini D, Blatt I, Amariglio N, Rechavi G, Tanne D, Chapman J. (2013) Thrombin regulation of synaptic plasticity: implications for physiology and pathology. Exp Neurol. 247:595-604.

(37) Stein ES, Itsekson-Hayosh Z, Aronovich A, Reisner Y, Bushi D, Pick CG, Tanne D, Chapman J, Vlachos A, Maggio N. (2015) Thrombin induces ischemic LTP (iLTP): implications for synaptic plasticity in the acute phase of ischemic stroke. Sci Rep. 5:7912.

(38) Davies BJ, Pickard BS, Steel M, Morris RG and Lathe R. (1998) Serine proteases in rodent hippocampus. J Biol Chem 273, 23004–23011.

(39) Yang Y, Zhang M, Kang X, Jiang C, Zhang H, Wang P, Li J. (2015) Impaired adult hippocampal neurogenesis and cognitive ability in a mouse model of intrastriatal hemorrhage. Neurosci Lett. 599:133-9.

(40) Armstrong DL, Garcia EA, Ma T, Quinones B, Wayner MJ. (1996) Angiotensin II blockade of long-term potentiation at the perforant path--granule cell synapse in vitro. Peptides.17(4):689-93.

(41) Wayner MJ, Armstrong DL, Polan-Curtain JL, Denny JB. (1993) Role of angiotensin II and AT1 receptors in hippocampal LTP. Pharmacol Biochem Behav. 45(2):455-64.

(42) Wayner MJ, Armstrong DL, Polan-Curtain JL, Denny JB. (1993) Ethanol and diazepam inhibition of hippocampal LTP is mediated by angiotensin II and AT1 receptors. Peptides. 14(3):441-4.

(43) Wayner MJ, Polan-Curtain J, Armstrong DL. (1994) Angiotensin AII AT1 receptor mediates ethanol-diazepam inhibition of

hippocampal LTP. Chin J Physiol. 37(2):55-61.

(44) Wayner MJ, Polan-Curtain J, Armstrong DL. (1995) Dose and time dependency of angiotensin II inhibition of hippocampal long-term potentiation. Peptides. 16(6):1079-82.

(45) Wayner MJ, Armstrong DL, Phelix CF. (1996) Nicotine blocks angiotensin II inhibition of LTP in the dentate gyrus. Peptides. 17(7):1127-33.

(46) Mukuda T, Koyama Y, Hamasaki S, Kaidoh T, Furukawa Y. (2014) Systemic angiotensin II and exercise-induced neurogenesis in adult rat hippocampus.Brain Res. 1588:92-103.

(47) Koyama Y, Mukuda T, Hamasaki S, Nakane H, Kaidoh T. (2018) Short-term Heat Exposure Promotes Hippocampal Neurogenesis via Activation of Angiotensin II Type 1 Receptor in Adult Rats. Neuroscience.385:121-132.

(48) Tancredi V, Zona C, Velotti F, Eusebi F, Santoni A. (1990) Interleukin-2 suppresses established long-term potentiation and inhibits its induction in the rat hippocampus. Brain Res. 525(1):149-51.

(49) Beck RD Jr, Wasserfall C, Ha GK, Cushman JD, Huang Z, Atkinson MA, Petitto JM. (2005) Changes in hippocampal IL-15, related cytokines, and neurogenesis in IL-2 deficient mice. Brain Res. 1041(2):223-30.

(50) Foy MR, Akopian G, Thompson RF. (2008) Progesterone regulation of synaptic transmission and plasticity in rodent hippocampus. Learn Mem. 15(11):820-2.

(51) Liu L, Wang J, Zhao L, Nilsen J, McClure K, Wong K, Brinton RD. (2009) Progesterone increases rat neural progenitor cell cycle gene expression and proliferation via extracellularly regulated kinase and progesterone receptor membrane components 1 and 2. Endocrinology. 150(7):3186-96.

(52) Zhang Z, Yang R, Zhou R, Li L, Sokabe M, Chen L. (2010) Progesterone promotes the survival of newborn neurons in the dentate gyrus of adult male mice. Hippocampus. 20(3):402-12.

(53) Pavlides C, Westlind-Danielsson AI, Nyborg H, McEwen BS. (1991) Neonatal hyperthyroidism disrupts hippocampal LTP and spatial learning. Exp Brain Res. 85(3):559-64.

(54) Taşkın E, Artis AS, Bitiktas S, Dolu N, Liman N, Süer C. (2011) Experimentally induced hyperthyroidism disrupts hippocampal long-term potentiation in adult rats. Neuroendocrinology. 94(3):218-27.

(55) Gilbert ME, Sui L. (2006) Dose-dependent reductions in spatial learning and synaptic function in the dentate gyrus of adult rats following developmental thyroid hormone insufficiency. Brain Res. 1069(1):10-22.

(56) Sui L, Anderson WL, Gilbert ME. (2005) Impairment in short-term but enhanced long-term synaptic potentiation and ERK activation in adult hippocampal area CA1 following developmental thyroid hormone insufficiency. Toxicol Sci. 85(1):647-56.

(57) Montero-Pedrazuela A, Venero C, Lavado-Autric R, Fernández-Lamo I, García-Verdugo JM, Bernal J, Guadaño-Ferraz A. (2006) Modulation of adult hippocampal neurogenesis by thyroid hormones: implications in depressive-like behavior. Mol Psychiatry. 11(4):361-71.

(58) Balschun D, Reymann KG. (1994) Cholecystokinin (CCK-8S) prolongs 'unsaturated' theta-pulse induced long-term potentiation in rat hippocampal CA1 in vitro. Neuropeptides.26(6):421-7.

(59) Yasui M, Kawasaki K. (1995) CCKB-receptor activation augments the long-term potentiation in guinea pig hippocampal slices. Jpn J Pharmacol. 68(4):441-7.

(60) Sui Y, Vermeulen R, Hökfelt T, Horne MK, Stanić D. (2013) Female mice lacking cholecystokinin 1 receptors have compromised neurogenesis, and fewer dopaminergic cells in the olfactory bulb. Front Cell Neurosci. 7:13.

(61) Reisi P, Ghaedamini AR, Golbidi M, Shabrang M, Arabpoor Z, Rashidi B. (2015) Effect of cholecystokinin on learning and memory, neuronal proliferation and apoptosis in the rat hippocampus.Adv Biomed Res. 4:227.

(62) Zhao M, Li D, Shimazu K, Zhou YX, Lu B, Deng CX. (2007) Fibroblast growth factor receptor-1 is required for long-term potentiation, memory consolidation, and neurogenesis. Biol Psychiatry. 62(5):381-90.

(63) Kang W, Hébert JM. (2015) FGF Signaling Is necessary for neurogenesis in young mice and sufficient to reverse its decline in old mice. J Neurosci. 35(28):10217-23.

(64) Chiang MY, Misner D, Kempermann G, Schikorski T, Giguère V, Sucov HM, Gage FH, Stevens CF, Evans RM. (1998) An essential role for retinoid receptors RARbeta and RXRgamma in long-term potentiation and depression. Neuron. 21(6):1353-61.

(65) Córdoba Montoya DA, Carrer HF. (1997) Estrogen facilitates induction of long-term potentiation in the hippocampus of awake rats. Brain Res. 778(2):430-8.

(66) Foy MR, Xu J, Xie X, Brinton RD, Thompson RF, Berger TW. (1999) 17beta-estradiol enhances NMDA receptor-mediated EPSPs and long-term potentiation. J Neurophysiol. 81(2):925-9.

(67) Bali N, Arimoto JM, Iwata N, Lin SW, Zhao L, Brinton RD, Morgan TE, Finch CE. (2012) Differential responses of progesterone receptor membrane component-1 (Pgrmc1) and the classical progesterone receptor (Pgr) to 17β-estradiol and progesterone in hippocampal subregions that support synaptic remodeling and neurogenesis. Endocrinology. 153(2):759-69.

(68) Hsu YT, Li J, Wu D, Südhof TC, Chen L. (2019) Synaptic retinoic acid receptor signaling mediates mTOR-dependent metaplasticity that controls hippocampal learning. Proc Natl Acad Sci U S A. 116(14):7113-7122.

(69) Boku S, Toda H, Nakagawa S, Kato A, Inoue T, Koyama T, Hiroi N, Kusumi I. (2015) Neonatal maternal separation alters the capacity of adult neural precursor cells to differentiate into neurons via methylation of retinoic acid receptor gene promoter. Biol Psychiatry. 77(4):335-44.

(70) Kapoor R, Ghosh H, Nordstrom K, Vennstrom B, Vaidya VA. (2011) Loss of thyroid hormone receptor β is associated with increased progenitor proliferation and NeuroD positive cell number in the adult hippocampus. Neurosci Lett. 487(2):199-203.

(71) Park SJ, Shin EJ, Min SS, An J, Li Z, Hee Chung Y, Hoon Jeong J, Bach JH, Nah SY, Kim WK, Jang CG, Kim YS, Nabeshima Y, Nabeshima T, Kim HC. (2013) Inactivation of JAK2/STAT3 signaling axis and downregulation of M1 mAChR cause cognitive impairment in klotho mutant mice, a genetic model of aging. Neuropsychopharmacology. 38(8):1426-37.

(72) Salech F, Varela-Nallar L, Arredondo SB, Bustamante DB, Andaur GA, Cisneros R, Ponce DP, Ayala P, Inestrosa NC, Valdés JL, Behrens MI, Couve A. (2017) Local Klotho enhances neuronal progenitor proliferation in the adult hippocampus. J Gerontol A Biol Sci Med Sci.

(73) Baratta MV, Lamp T, Tallent MK. (2002) Somatostatin depresses long-term potentiation and Ca2+ signaling in mouse dentate gyrus. J Neurophysiol. 88(6):3078-86.

(74) Fan W, Fu T. (2014) Somatostatin modulates LTP in hippocampal CA1 pyramidal neurons: differential activation conditions in apical and basal dendrites. Neurosci Lett. 561:1-6.

(75) Skucas VA, Duffy AM, Harte-Hargrove LC, Magagna-Poveda A, Radman T, Chakraborty G, Schroeder CE, MacLusky NJ, Scharfman HE. (2013) Testosterone depletion in adult male rats increases mossy fiber transmission, LTP, and sprouting in area CA3 of hippocampus.J Neursci. 33(6):2338-55.

(76) Pettorossi VE, Di Mauro M, Scarduzio M, Panichi R, Tozzi A, Calabresi P, Grassi S. (2013) Modulatory role of androgenic and estrogenic neurosteroids in determining the direction of synaptic plasticity in the CA1 hippocampal region of male rats. Physiol Rep. 1(7):e00185.

(77) Di Mauro M, Tozzi A, Calabresi P, Pettorossi VE, Grassi S. (2017) Different synaptic stimulation patterns influence the local androgenic and estrogenic neurosteroid availability triggering hippocampal synaptic plasticity in the male rat.Eur J Neurosci. 45(4):499-509.

(78) Galea LA. (2008) Gonadal hormone modulation of neurogenesis in the dentate gyrus of adult male and female rodents. Brain Res Rev. 57(2):332-41.

(79) Hamson DK, Wainwright SR, Taylor JR, Jones BA, Watson NV, Galea LA. (2013) Androgens increase survival of adult-born neurons in the dentate gyrus by an androgen receptor-dependent mechanism in male rats. Endocrinology. 154(9):3294-304.

(80) Swift-Gallant A, Duarte-Guterman P, Hamson DK, Ibrahim M, Monks DA, Galea LAM. (2018) Neural androgen receptors affect the number of surviving new neurones in the adult dentate gyrus of male mice. J Neuroendocrinol. 30(4):e12578.
